# Multifaceted DNA metabarcoding: Validation of a noninvasive, next‐generation approach to studying bat populations

**DOI:** 10.1111/eva.12644

**Published:** 2018-05-31

**Authors:** Joel F. Swift, Richard F. Lance, Xin Guan, Eric R. Britzke, Denise L. Lindsay, Christine E. Edwards

**Affiliations:** ^1^ Center for Conservation and Sustainable Development Missouri Botanical Garden St. Louis Missouri; ^2^ Environmental Laboratory US Army Engineer Research and Development Center Vicksburg Mississippi; ^3^ Bennett Aerospace Cary North Carolina

**Keywords:** bats, DNA metabarcoding, next‐generation DNA sequencing, noninvasive sampling, population assessment

## Abstract

As multiple species of bats are currently experiencing dramatic declines in populations due to white‐nose syndrome (WNS) and other factors, conservation managers have an urgent need for data on the ecology and overall status of populations of once‐common bat species. Standard approaches to obtain data on bat populations often involve capture and handling, requiring extensive expertise and unavoidably resulting in stress to the bats. New methods to rapidly obtain critical data are needed that minimize both the stress on bats and the spread of WNS. Guano provides a noninvasive source of DNA that includes information from the bat, but also dietary items, parasites, and pathogens. DNA metabarcoding is a high‐throughput, DNA‐based identification technique to assess the biodiversity of environmental or fecal samples. We investigated the use of multifaceted DNA metabarcoding (MDM), a technique combining next‐generation DNA sequencing (NGS), DNA barcodes, and bioinformatic analysis, to simultaneously collect data on multiple parameters of interest (bat species composition, individual genotype, sex ratios, diet, parasites, and presence of WNS) from fecal samples using a single NGS run. We tested the accuracy of each MDM assay using samples in which these parameters were previously determined using conventional approaches. We found that assays for bat species identification, insect diet, parasite diversity, and genotype were both sensitive and accurate, the assay to detect WNS was highly sensitive but requires careful sample processing steps to ensure the reliability of results, while assays for nectivorous diet and sex showed lower sensitivity. MDM was able to quantify multiple data classes from fecal samples simultaneously, and results were consistent whether we included assays for a single data class or multiple data classes. Overall, MDM is a useful approach that employs noninvasive sampling and a customizable suite of assays to gain important and largely accurate information on bat ecology and population dynamics.

## 1 INTRODUCTION

With animal species increasingly facing threats to their persistence from changing climates, disease, habitat loss, and other pressures from human activities, scientists are observing accelerated rates of extinction (Barnosky et al., 2011; Ceballos et al., 2015; IPCC, 2007). For effective conservation, it is vital for land managers to have accurate information about the status and dynamics of natural populations of animal species. Using conventional techniques, collection of the data needed to inform conservation efforts requires teams with specialized field expertise, may involve capture and handling of the animals, and typically results in stress to the target species. In recent times, new approaches and technologies have been developed that greatly advance our ability to conduct assessments of wild animal populations in a noninvasive manner.

Noninvasive sampling (NIS), which involves sampling individuals indirectly by collecting biological materials left in the environment, such as eggshells, feathers, saliva, hairs, urine, or feces, is increasingly being employed to conduct assessments of animal populations, eliminating the need to capture or handle an animal (Beja‐Pereira, Oliveira, Alves, Schwartz, & Luikart, 2009) and vastly reducing the stress involved (Arandjelovic et al., 2010; Eggert, Eggert, & Woodruff, 2003; Rudnick, Katzner, Bragin, Rhodes, & Dewoody, 2005; Steyer, Simon, Kraus, Haase, & Nowak, 2013). NIS is often preferred when working with species that are difficult to capture, that are physiologically sensitive or may modify behavior in unwanted ways in response to capture, or that pose a threat to the collector. One of the most common ways that NIS samples are used to conduct population assessments is through the analysis of DNA in the sample, which can provide information about the genotype of a target individual as well as data on population parameters such as genetic diversity, population size, and population structure (Adams, Kelly, & Waits, 2003; Bellemain, Swenson, Tallmon, Brunberg, & Taberlet, 2005; Fernando, Pfrender, Encalada, & Lande, 2000; Flagstad et al., 2004).

Of all of the types of samples collected using NIS, fecal samples are one of the most commonly utilized types of NIS samples because of their ease of collection (Waits & Paetkau, 2005). Another benefit of fecal samples is that they contain DNA not only of the target individual but also from the environment, dietary items, parasites, and gut microbiota of the individual. The nontarget DNA present in fecal samples may provide a wealth of information about a population, and new approaches have been developed to utilize this additional information (Taberlet, Coissac, Pompanon, Brochmann, & Willerslev, 2012). This approach, called DNA metabarcoding, leverages the power of new, high‐throughput DNA sequencing technologies (i.e., next‐generation sequencing; NGS), reliable molecular markers (i.e., DNA barcodes), extensive DNA barcode databases (i.e., GenBank, BOLD, and EMBL), and advancements in statistical analysis. In DNA metabarcoding, PCR is conducted using genetic markers (i.e., DNA barcodes) that target specific taxonomic groups, the PCR products are sequenced using NGS, and the data are compared to DNA sequence databases to categorize and annotate the diversity of DNA sequences in a sample (Huson et al., 2016; Pompanon et al., 2012; Taberlet et al., 2012).

DNA metabarcoding of fecal samples (i.e., molecular scatology) has been employed to understand several types of data in mammalian taxa, including the genotype of the individual (De Barba et al., 2017), its diet (De Barba et al., 2014; Quemere et al., 2013; Shehzad et al., 2012; Soininen et al., 2013; Zeale, Butlin, Barker, Lees, & Jones, 2011), and its pathogens/parasites (Aivelo, Laakkonen, & Jernvall, 2016; Aivelo, Medlar, Löytynoja, Laakkonen, & Jernvall, 2015; Springer et al., 2017). While most previous studies generally investigated only a single attribute of a population, combining assays for multiple classes of data for a species in a metabarcoding study (i.e., multifaceted DNA metabarcoding; MDM) would likely be an efficient approach to gain an in‐depth picture of the diverse ecological interactions of a target species. However, whether it is possible to simultaneously obtain accurate data for multiple data classes from small and degraded fecal DNA samples is a remaining challenge.

Bats play an essential role in the functioning of ecosystems and provide a multitude of vital ecological services, such as insect predation, pollination, seed dispersal, and nutrient cycling (Kasso & Balakrishnan, 2013; Kunz, de Torrez, Bauer, Lobova, & Fleming, 2011; Maine & Boyles, 2015). In the United States, many bat species currently face threats from both biological and anthropogenic sources. For example, white‐nose syndrome (WNS), a disease linked to the fungal pathogen *Pseudogymnoascus destructans* (*Pd*; formerly *Geomyces destructans*), has caused up to >90% overall declines in populations of some species (Courtin, Stone, Risatti, Gilbert, & Van Kruiningen, 2010; Lorch et al., 2011). These declines in bat species have resulted in increased conservation interest in species that were, until recently, very abundant; however, conservation actions are often limited by a lack of data for these species. Conventional approaches for collecting necessary population information on bats often involve handling bats, which requires expertise to ensure the safety of the bat and the integrity of the roost and may involve risk of injury/stress to bats or spread of *Pd* spores through contaminated equipment (Foley, Clifford, Castle, Cryan, & Ostfeld, 2011; USFWS, 2016). Thus, the development and demonstration of methods that minimize the risks involved in collecting data on bats (and other taxa) are desirable.

In this study, we designed a DNA metabarcoding protocol, multifaceted DNA metabarcoding (MDM), which uses NIS and DNA metabarcoding to generate multiple conservation‐relevant classes of population data from bat guano samples. The strategy for developing MDM was to develop an assay for each data class by collecting bat fecal samples from individuals or populations where the identity of the data class was known and using several PCR primer pairs and NGS sequencing of a single data class to identify the markers that had the greatest accuracy to recover the known population parameters. We then combined PCR products from all six data class into one large MDM run. Our objectives were (i) to test the accuracy of MDM assays in characterizing bat species, sex, parasite diversity, presence of *Pd*, diet composition, and individual genotypes, and (ii) to assess the potential utility of using MDM to gather information for multiple types of data from a large set of guano samples simultaneously in a single NGS run.

## 2 METHODS AND MATERIALS

### 2.1 Overall experimental design

**Figure 1:**
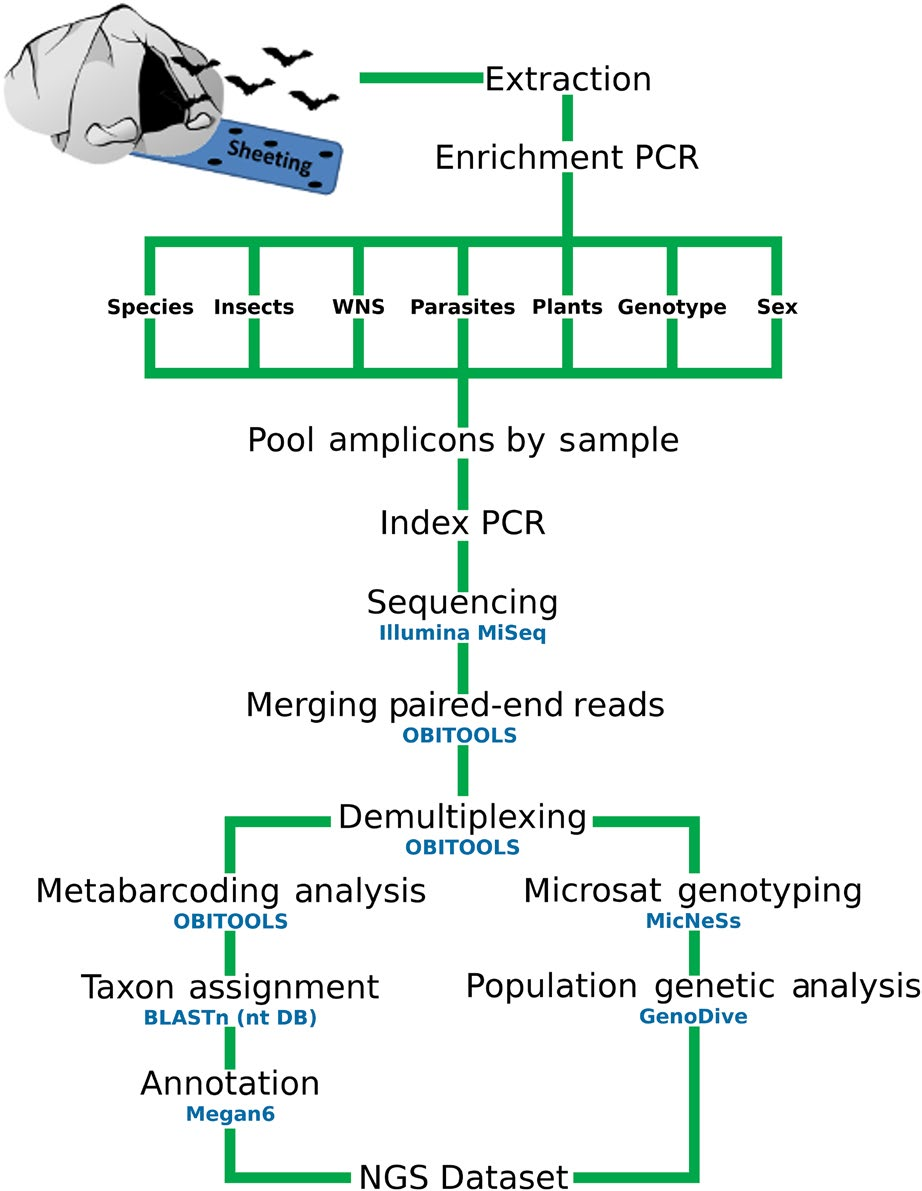
Workflow schematic outlining the experimental design of the multifaceted DNA metabarcoding (MDM) approach to analyze bat guano

The strategy for developing MDM was to collect bat fecal samples from individuals or populations where the identity of specific data classes (i.e., bat species identification, sex identification, microsatellite genotype, dietary items for both nectivorous and insectivorous bats, endoparasite diversity, and presence of *Pd*) was known (Table 1 and Supporting Information Table S1). We then conducted PCR of these samples using barcoding markers that targeted a specific data class (Table 2 and Supporting Information Table S2), sequenced the PCR products from a single data class using NGS, and analyzed the data to determine the accuracy of each marker to recover the data class of interest. The most accurate PCR primers for each data class were then selected for the overall combined MDM protocol (Figure 1). In the overall MDM run, we combined the PCR products from multiple assays and multiple samples, sequenced the combined amplicon pools using NGS, and evaluated whether multiple primers sets and data classes could be combined successfully and produce comparable results to the NGS runs that contained markers for only a single data class.

**Table 1:** Summary of the validation methods, bat species, and number of individuals (*n*) used to test the accuracy of each assay. Supporting Information Table S1 for detailed collection information

Assay	Validation method	Test species	*n* (NGS)	*n* (MDM)
Insectivorous diet	*Sanger, NGS, MDM*	*Antrozous pallidus* LeConte	42	8
Nectivorous diet	*Sanger, NGS, MDM*	*Leptonycteris yerbabuenae* Martínez and Villa‐R.	42	8
Detection of *Pseudogymnoascus destructans*	*qPCR, MDM*	*Myotis lucifugus* LeConte	–	8
Endoparasite diversity	*NGS, MDM*	*Eptesicus fuscus* Palisot de Beauvois	16	8
Species identification	*Sanger, MDM*	*A. pallidus*	–	8
		*Corynorrhinus rafinesquii* Lesson		24
		*E. fuscus*		8
		*L. yerbabuenae*		8
		*M. lucifugus*		8
Individual genotype	*Fragment analysis, NGS, MDM*	*C. rafinesquii*	94	24
Sex identification	*Gel visualization, NGS*	*E. fuscus*	10	–
		*Lasiurus borealis* Müller	10	
		*M. austroriparius* Rhoads	10	
		*M. grisescens* Howell	10	
		*M. lucifugus*	10	
		*M. sodalis* Miller and Allen	10	
		*Nycticeius humeralis* Rafinesque	10	

**Table 2:** The optimal primers selected for each data class based on accuracy

Target group	Forward Primer	Forward primer sequence	Reverse Primer	Reverse primer sequence	Region	Size (bp)	Citation
Insects	ZBJ‐ArtF1c	AGATATTGGAACWTTATATTTTATTTTTGG	ZBJ‐ArtR2	WACTAATCAATTWCCAAATCCTCC	*COI*	157	(Zeale et al., 2011)
Insects/Bat species ID	Ins16S_1_F	TRRGACGAGAAGACCCTATA	Ins16S_1_R	TCTTAATCCAACATCGAGGTC	*16s*	216	(Clarke et al., 2014)
Plants	psbAF	GTTATGCATGAACGTAATGCTC	Trn‐HR2	CGCGCATGGTGGATTCACAAT	*TrnH‐psbA*	185–887	(Sang, Crawford, & Stuessy, 1997)(Kress, Wurdack, Zimmer, Weigt, & Janzen, 2005)
Endoparasites	MN18F	CGCGAATRGCTCATTACAACAGC	22R reverse	GCCTGCTGCCTTCCTTGGA	*18s rDNA*	345	(Bhadury et al., 2006)
*P. destructans*	nu‐IGS‐0169‐5′	TGCCTCTCCGCCATTAGTG	nu‐IGS‐0235‐3′	ACCACCGGCTCGCTAGGTA	Fungal *IGS*	114–310	(Muller et al., 2013)
Sex primer	KXZF‐F	AGTCAAGGGRTGTCCATCR	KXZF‐R	GTTTGYASACCAGGTTCCTC	Zinc finger X	245	(Korstian et al., 2013)
Sex primer	KYZF‐F	GGTRAGDGCACAYRAGTTCCACA	KYZF‐R	TGCYATTACAAAACCTT	Zinc finger Y	80	(Korstian et al., 2013)
Sex primer	XGYC‐F	GCTGCTAAGCCACATATAGCT	XGYC‐R	CCTGAATGTCTGTTCCAAAGACG	Zinc finger Y	121	(Lance et al., unpublished data)
Sex primer	XGXC‐F	TGCGAGCTCTCAGATGAAACT	XGXC‐R	TCCCTGTTCAATCCATTCCGT	Zinc finger X	174	(Lance et al., unpublished data)
*Corynorrhinus* Microsatellite	Cora_E07F	TTACTAAAGGTTTGGGTAGGGAA	Cora_E07R	GTGAAGTAGCCTGGCCTAAGA	Nuclear DNA	163–179	(Piaggio et al., 2009)
*Corynorrhinus* Microsatellite	Cora_C07	CATTGGCTTTGTCTTAACAATTT	Cora_G01	TTTGTTTCAGTTTCTCTCTCTCC	Nuclear DNA	191–213	(Piaggio et al., 2009)
*Corynorrhinus* Microsatellite	Cora_F02F	GTCACTGGCTACAAAGAATGAAG	Cora_F02R	GAAACACAGCAGAATTGTCTCTC	Nuclear DNA	201–263	(Piaggio et al., 2009)
*Corynorrhinus* Microsatellite	Cora_G10	TTACAGTAGATACGGTTGTGCCT	Cora_A08	TTTTAGGACTGGTTTTAGGGAAG	Nuclear DNA	259–277	(Piaggio et al., 2009)
*Corynorrhinus* Microsatellite	Cora_B07	TTAGACAAATGAGGGAGGATTG	Cora_H12	CATCAAAGAATGCCAAACTAAAG	Nuclear DNA	271–313	(Piaggio et al., 2009)
*Corynorrhinus* Microsatellite	Cora_E10	ACTTTTCATTCTTTCCCATTCT	Cora_ G03	AAACCAACGAGTGCTAAATCTAC	Nuclear DNA	333–357	(Piaggio et al., 2009)

### 2.2 Sample collection and methods for the development of assays for each data class

Prior to conducting research, we obtained Institutional Animal Care and Use Committee (IACUC EL‐FR‐2014‐1) approval from the Environmental Laboratory of the United States Army Engineer Research and Development Center. We also obtained all necessary state and federal permits prior to sample collection. Fecal samples were collected from bat species from across the United States, including *Eptesicus fuscus, Leptonycteris yerbabuenae, Antrozous pallidus, Lasiurus borealis, Myotis austroriparius, M. grisescens, M. lucifugus, M. sodalis, Nyctieius humeralis, and Corynorhinus rafinesquii* (See Table 1 and Supporting Information Table S1 for a detailed description of all samples used in the study). We also collected information about specific data classes using conventional techniques to determine the accuracy of corresponding results obtained using molecular markers.

#### 2.2.1 Diet analysis

To test the accuracy of markers for diet analysis in both insectivorous and nectivorous bat species, we provided bats within an enclosure at the Fort Worth Zoo with selected diet items. Two *A. pallidus* (insectivorous) bats were presented with eleven insect species from four orders (Carolina Biological Supply, Burlington, NC, USA; Table 3) and allowed to feed ad libitum on preferred items. Eleven *L. yerbabuenae* (nectivorous) bats were presented with pollen from seven plant species from four plant families (Table 3; The Pollen Bank, Bakersfield, CA, USA), mixed with Roudybush Nectar 3 (Woodland, CA, USA), which has a soy protein base (*Glycine max;* Fabaceae). Supporting Information Table S3 for the exact composition of the nectivorous controlled feeding. Bats were provided the controlled diet items starting 1 week prior to sample collection to ensure that previously ingested materials were passed. Although the bats were within an enclosure, insects other than the provided diet items may still have been present due to infiltration from the external environment.

**Table 3:** Diet items offered to the insectivorous and nectivorous bats in the controlled feeding trials at Fort Worth Zoo

Insectivorous		
Common name	Order	Identity
Red flour beetle	Coleoptera	*Tribolium castanem*
Meal worm	Coleoptera	*Tenebrio molitor*
Rice flour beetle	Coleoptera	*Tribolium confusum*
Bean beetle	Coleoptera	*Callosobruchus maculatus*
Solider fly	Diptera	*Hermetia illucens*
Springtail	Diptera	*Calliphora vomitoria*
Large flighted fruit fly	Diptera	*Drosophila hydei*
Butterworm	Lepidoptera	*Chilecomadia sp*.
Silkworm	Lepidoptera	*Bombyx mori*
Hornworm	Lepidoptera	*Manduca sexta*
Cricket	Orthoptera	*Acheta domesticus*

Nectivorous		
Common name	Family	Identity
Soy	Fabaceae	*Glycine max*
Kiwi	Actinidiaceae	*Actinidia chinensis*
Date	Arecaceae	*Phoenix dactylifera*
Olive	Oleaceae	*Olea europaea*
Apple	Rosaceae	*Malus pumila*
Plum	Rosaceae	*Prunus salicina*
Cherry	Rosaceae	*Prunus avium*
Almond	Rosaceae	*Prunus dulcis*

Guano was collected over the course of 4 days by placing disposable plastic sheeting in the bat enclosure. Sheeting was left overnight, and guano samples were collected from the sheeting. Bat fecal matter generally came in two forms: solid guano pellets and “splats,” or liquid stool (produced by nectivorous bats). For the solid pellets, we used sterile, single‐use tweezers to place each pellet into an individual tube containing silica gel desiccant. Samples were stored at room temperature in cardboard boxes (to reduce potential light‐induced DNA degradation) until DNA extraction. Each “splat” was collected using a sterile single‐use swab and then placed in an individual tube filled with 500 μl of cetyltrimethyl ammonium bromide (CTAB) solution. Samples were kept on ice in a cooler in transit to the laboratory, where they were stored at −20°C until DNA extraction. Negative control samples, where we swabbed the blank sheeting, were also collected. DNA was extracted from the fecal samples as described below (see Molecular Methods below).

We first tested the accuracy of PCR primers (Supporting Information Table S2) to identify each diet item individually (i.e., from direct extractions of each diet item) by conducting PCR, Sanger sequencing (see Supporting Information Appendix S1 for protocol), and BLAST searches of the resulting sequences. For insect diet items, we initially tested two markers: *16s* rRNA and cytochrome oxidase 1 (*COI*; see Table 2 for primer information). For the plant diet items, we initially tested nine PCR primer pairs covering the six most common universal barcode markers for plants (*ITS1, ITS2, rbcL, matK, trnL‐F,* and *trnH‐psbA*; Tables 2 and Supporting Information Table S2). The two insectivorous diet and nine nectivorous diet primers were also used in an initial NGS test (see Molecular Methods below) to assess the diet in 42 fecal samples each of *A. pallidus* and *L. yerbabuenae* from the controlled feeding runs at the Fort Worth Zoo (Table 1 and Supporting Information Table S1). Positive controls, consisting of directly extracted samples of each diet item pooled equimolar in ratios (i.e., the insects provided to the bats or the pollen added in the nectar mix), were tested in NGS trials alongside the guano samples. We then selected the two insect diet primers (Table 2 and Supporting Information Table S2) and four plant diet primers targeting *ITS2, rbcL* (primers 1 and 724R)*, trnL‐F* (primers E and F)*,* and *trnH‐psbA* (Table 2 and Supporting Information Table S2) for the combined MDM run to analyze guano samples.

#### 2.2.2 Pd detection

To test the accuracy of PCR primers to detect *Pd*, we collected fecal material from the intestinal tracts of previously sacrificed specimens of *M. lucifugus* that tested positive for WNS at the United States Geological Survey’s National Wildlife Health Center (USGS NWHC; Madison, WI, USA; Supporting Information Table S2). We first tested the accuracy of the *Pd*‐specific real‐time quantitative PCR (qPCR) primers developed by Muller, Lorch, Lane, and Gargas (2013) to amplify *Pd* from the fecal material from each WNS‐positive bat using the sample processing and qPCR protocols described in Supporting Information Appendix S1. For MDM, we used the primers from the qPCR assay to amplify *Pd* (Table 2) in eight of the WNS‐positive fecal samples of *M. lucifugus,* as well as 48 other bats (*E. fuscus*,* C. rafinesquii*,* L. yerbabuenae*, and *A. pallidus*), which were considered as negative controls, as they were asymptomatic for WNS (Table 1) and collected in locales and seasons where WNS is not present. The *Pd* assay was then sequenced and validated directly in the MDM run.

#### 2.2.3 Parasite analysis

For the endoparasite analysis, fecal samples were collected from 16 *E. fuscus* from a single population on the United States Army Crane Army Ammunition Activity (Crane, IN, USA), which were then euthanized and submitted for endoparasite necropsy analysis at the University of California, Davis, College of Veterinary Medicine. The necropsies involved searching for endoparasites in the lungs, livers, and mucosa of the intestinal tract. Parasites were then collected, stored in 70% ethanol, and identified using microscopy to the lowest taxonomic level possible. Using NGS, we tested eight PCR primer pairs for nematode and trematode endoparasites targeting *COI* and *18s* rRNA (Tables 2 and Supporting Information Table S2). *18s* rRNA provided resolution to the family and genus level in previous studies of Nematoda (Bhadury et al., 2006) and Trematoda (Moszczynska, Locke, McLaughlin, Marcogliese, & Crease, 2009), and *COI* provided species‐level resolution in certain nematode linages (Derycke et al., 2008; Moszczynska et al., 2009). Accuracy was assessed by comparing the NGS results to those from the necropsy analysis. We then selected the most accurate primer pair for MDM.

#### 2.2.4 Species identification

To test the accuracy of bat species identification (ID) markers, we used guano samples of 56 previously identified individuals from five species of bats (*A. pallidus, C. rafinesquii, E. fuscus, L. yerbabuenae, and M. lucifugus*) that were originally collected for testing other data classes (Table 1 and Supporting Information Table S1). We tested five primer pairs targeting *16s* rRNA and *COI* (Tables 2 and Supporting Information Table S2) in the full MDM run and determined their accuracy by comparing results to those of the known species identification.

#### 2.2.5 Sex determination

To test for accuracy in sex ID markers, we used guano samples collected from 70 individuals of seven bat species that were captured and visually inspected to determine sex (Table 1 and Supporting Information Table S1). We used both previously developed and newly designed primer cocktails, both of which were assayed using both gel electrophoresis and MDM. We initially tested the primer cocktail described by Korstian, Hale, Bennett, and Williams (2013), but they demonstrated poor or no amplification for some species, so we designed and tested an additional primer cocktail for sex ID, XGXYC (Table 2; Lance, Guan, & Piaggio, unpublished data). We scored the sex of each sample by observing PCR products on 2% agarose precast E‐gels (Thermo Fisher) stained with ethidium bromide. Both the Korstian and XGXYC primer cocktails (Table 2) are made up of two primers that target the X or Y chromosome; males produce two bands, one from an X‐chromosome locus and the other from a Y‐chromosome locus, while females should produce only a single band from the X‐chromosome locus. The X‐chromosome band acts as an internal control as it should be present within any sample with sufficient DNA for amplification (Shaw, Wilson, & White, 2003).

For NGS analysis, we used both the Korstian and XGXYC primer cocktails (Table 2), as each successfully amplified in only a subset of bat taxa. Results were compared to the known sex of the individual and scored as follows: Samples that did not produce hits to either the X or Y chromosome were labeled as “failed,” female samples that produced inaccurate hits to the Y chromosome or male samples that did not produce hits to the Y chromosome were labeled as “misidentified,” and samples that produced hits to only the X chromosome in females and both the X and Y chromosomes in males were labeled as “identified.”

#### 2.2.6 Genotype analysis

To determine whether microsatellites obtained from bat guano samples could be effectively characterized through NGS, we compared NGS data to that obtained for the same markers and samples using conventional fragment analysis protocols. A total of 94 *C. rafinesquii* guano samples (Table 1 and Supporting Information Table S1) were collected in 2014 from sheeting placed underneath an artificial roost structure at Mammoth Cave National Park, KY, USA. Samples were genotyped at 14 previously developed microsatellite loci (Piaggio, Figueroa, & Perkins, 2009) using a conventional fragment analysis genotyping approach (Supporting Information Appendix S1 for a description of the conventional approach). We conducted an initial NGS run with the same 14 microsatellite loci for 71 *C. rafinesquii*. Six of the loci did not amplify consistently using either the conventional or NGS approaches and were removed from all subsequent analyses. To determine repeatability of the NGS approach, we used the eight microsatellite loci to genotype two replicates of each of an additional 23 samples of *C. rafinesquii*. Although we also included 24 individuals of *C. rafinesquii* and the eight microsatellite loci in the MDM run, we tested a different type of *Taq* in the microsatellite enrichment PCR for the MDM run, such that these results are not directly comparable to those of the NGS run. NGS/MDM data were analyzed as described in “Microsatellite Scoring and Analysis” below.

#### 2.2.7 Summary of NGS and MDM runs

In summary, we conducted five unique NGS runs that targeted a single data class, including (i) eight primers that targeted endoparasites in 16 samples of *E. fuscus* (Tables 1 and 2, Supporting Information Tables S1 and S2), (ii) eleven primers for diet, two of which targeted insectivorous diet (*COI* and *16s*) and nine that targeted nectivorous diet items (Tables 1 and 2, Supporting Information Tables S1 and S2), from 42 fecal samples each of *A. pallidus* and *L. yerbabuenae* from the controlled feedings at the Fort Worth Zoo*,* (iii) fourteen microsatellite loci to genotype 71 *C. rafinesquii* fecal samples, (iv) eight microsatellite loci for 23 samples of *C. rafinesquii*, which were replicated to determine repeatability, and 5) four primers for sex identification that were amplified in 10 samples each of seven species: *E. fuscus, L. borealis, M. austroriparius, M. grisescens, M. lucifugus, M. sodalis, and N. humeralis*. We ran each of these data classes independently to ensure good coverage depths (Table 1).

We also conducted a full MDM run including all six data classes (Table 1), which was used to test whether we could obtain data from multiple data classes and multiple samples from a single NGS run. The primers employed in the MDM run were as follows: two for insectivorous diet, four for nectivorous diet, one for *Pd*, one for endoparasites, five for bat species ID, four for sex ID, and eight microsatellite loci for *C. rafinesquii* (Table 2 and Supporting Information Table S2). These primers were tested in a set of 56 guano samples from five bat species (Table 1), including eight of both *A. pallidus* and *L. yerbabuenae* (from the diet analysis), eight *E. fuscus* (from the endoparasite analysis), eight *M. lucifugus* that were positive for WNS, and 24 *C. rafinesquii* (from the microsatellite genotype analysis). For two data classes (bat species identification and *Pd* detection; Table 1), we did not conduct a single‐assay NGS run and the MDM run was used directly to validate their accuracy. To determine whether the results obtained using MDM provided comparable sensitivity and accuracy to single‐assay NGS, we tested the same samples and primers using NGS and MDM for the insectivorous and nectivorous diet and endoparasite assays.

### 2.3 Molecular methods and data analysis

#### 2.3.1 Molecular methods

DNA was extracted from each sample using a CTAB protocol (Doyle & Doyle, 1987), which was modified using smaller lysis and wash volumes and adding an additional wash step with 95% ethanol. DNA concentrations were quantified using a Qubit v.2 with the dsDNA HS assay kit (Thermo Fisher Scientific). We initially selected PCR primers from previous barcoding and metabarcoding studies that targeted relevant taxa or data classes (see especially Pompanon et al., 2012; Clarke, Soubrier, Weyrich, & Cooper, 2014; Brandon‐Mong et al., 2015; Table 2 and Supporting Information Table S2). All primers were first tested for PCR amplification success by observing PCR products on agarose gels; primers with poor amplification were eliminated.

To avoid contamination, all PCR steps (see below) were conducted in a sterile laminar flow hood that was physically separated from locations where DNA extraction or post‐PCR sample processing occurred; hood surfaces were sterilized with a 10% bleach solution and then treated with ultraviolet light for 15 min prior to PCR preparation. We included a negative control at each step of the protocol, including extraction and PCR steps.

The enrichment PCR and library preparation for NGS followed the Illumina^®^ 16s metagenomic protocol (Illumina, 2013) with some modifications and involves two rounds of PCR, each followed by a cleanup step. The initial enrichment PCR amplifies a barcode marker targeting a specific data class and also adds an overhang to enable the addition of an index and Illumina adapter in a subsequent round of PCR. These PCRs were conducted in 25 μl reactions containing 12.5 μl of 2 × KAPA HotStart ReadyMix (KAPA Biosystems), 1 μl of DNA, and 4 pmol each of the Forward (F) and Reverse (R) primers. All primers utilized for the first‐round PCR included a 5′ overhang to enable indexing in the next PCR step (Forward overhang: 5′ TCGTCGGCAGCGTCAGATGTGTATAAGAGACAG [locus‐specific sequence], reverse overhang: 5′ GTCTCGTGGGCTCGGAGATGTGTATAAGAGACAG [locus‐specific sequence]). PCR temperature cycling conditions were as follows: (i) 3 min at 94°C, (ii) denaturation for 30 s at 94°C, (iii) annealing for 30 s at 52°C, (iv) extension for 45 s at 72°C, (v) 34 repetitions of steps 2–4, and (vi) a final elongation at 72°C for 10 min. After enrichment, each sample was cleaned using AMPure XP beads (Beckman Coulter) following the manufacturer’s standard protocol, using a bead:DNA ratio of 1:1.6.

For the second PCR, amplicons for each sample from the different PCR primers were pooled and amplified using the Nextera index kit (Illumina^®^) to add sample‐specific indices and Illumina^®^ sequencing adapters. We quantified the cleaned PCR products and pooled 110 ng of each PCR product into a sample‐specific, combined amplicon pool. The second PCR volume was 50 μl and was comprised of 25 μl of 2 × KAPA HotStart ReadyMix, 5 μl of the sample amplicon pool, and 5 μl of each of two indices from the Nextera index kit (Illumina^®^). PCR temperature cycling conditions were as follows: (i) 3 min at 95°C, (ii) denaturation for 30 s at 95°C, (iii) annealing for 30 s at 55°C, (iv) extension for 30 s at 72°C, (v) eight repetitions of steps 2–4, and (vi) a final elongation at 72°C for 5 min. Samples were cleaned as specified above, quantified, normalized, pooled, and sequenced using 2 × 300 bp reads on an Illumina^®^ MiSeq.

#### 2.3.2 Data processing

The data processing approach for the microsatellite data differs from the remaining data classes and is presented under the “Microsatellite Scoring and Analysis” subheading below. The following protocols describe the processing and analysis of all other data classes. Reads were processed and filtered using OBITOOLS v1.01, a python‐based set of programs designed for analyzing DNA metabarcoding data (Boyer et al., 2016). In brief, paired‐end reads were merged with the illuminapairedend function using a minimum score of 40. When the score minimum was not reached, the reads were concatenated. Samples were then demultiplexed by marker sequentially using ngsfilter. ngsfilter is normally used for DNA fragments that include both a tag and primer, but the sequences in our study did not include a tag; we therefore developed a protocol to use ngsfilter for sequences without a tag, which is described in the Github (see “Data Archiving Statement” below). In brief, we assigned sequences to a marker by requiring a match to both the F and R primers, then by requiring that they match only the F primer; reads with no F primer match were passed to a separate file in which the process above was repeated for each marker. Reads for each PCR primer pair were then collapsed into unique entries using obiuniq and filtered based on the length of the sequence and read depth with obigrep.

Taxonomic assignment of sequences was conducted using BLASTN, the basic local alignment search tool for nucleotide sequences (blastn‐2.2.31+; Camacho et al., 2009). For the bat species, parasite diversity, *Pd*, and diet assays, we performed local alignments of query sequences to the NCBI nt database (downloaded 09/21/2016; NCBI Resource Coordinators, 2017); BLAST searches were run in parallel using GNU parallel (Tange, 2011). For sex identification, reads were annotated by conducting BLASTN searches against a custom database composed of sequences of the DNA regions generated using the Korstian and XGXYC primers. Taxonomic binning was performed on output files using the default settings in MEGAN6 CE v6.5.8 with the naive lowest common ancestor (LCA) algorithm (Huson et al., 2016). MEGAN6 filters out hits with poor e‐values and percent identities and then summarizes the taxonomic hits to a query and finds the “lowest common ancestor” or lowest taxonomic rank common to the hits, such that each read has only one assignment at the most appropriate taxonomic rank. For bat species identification, we extracted the 10 hits with the highest read counts for each sample to identify the bat species; we removed samples that did not have a match to a Chiropteran species with a threshold e‐value of 1E‐100 or that were assigned to more than one Chiropteran species.

#### 2.3.3 Microsatellite scoring and analysis

For microsatellite data generated using NGS, data were initially processed using OBITOOLS; paired‐end reads were merged with the illuminapairend function, and unmerged reads were removed. Data were demultiplexed using ngsfilter. Sequences were then converted to FASTA format and scored using MicNeSs v1.1 (Suez et al., 2016), which allows for the automation of the microsatellite scoring process from NGS data, which is necessary given the large numbers of sequences generated using this experimental design. MicNeSs scans the files, extracts all microsatellites above a specified number of repeats, and determines the repeat motif based on the microsatellite with the greatest number of observed repeats. The number of repeats is scored by building a distribution for each allele and then selecting the most frequent repeat number as the first allele. Datasets generated using the conventional and MDM approaches were each analyzed to quantify missing data. Population genetic summary statistics were analyzed in Genodive v2.0b23 (Meirmans & Van Tienderen, 2004), and the number of individuals present in the population was calculated using identity analysis in Cervus v3.0.7 (Kalinowski, Taper, & Marshall, 2007).

## 3 RESULTS

### 3.1 Overall results of the NGS runs

In this study, we conducted six NGS runs, five of which targeted a single data class, and one of which was a MDM run targeting all six data classes. The overall read count for all six of these runs ranged from 2,820,973 to 24,105,553 (mean = 13,349,653).

### 3.2 Diet analysis

**Figure 2:**
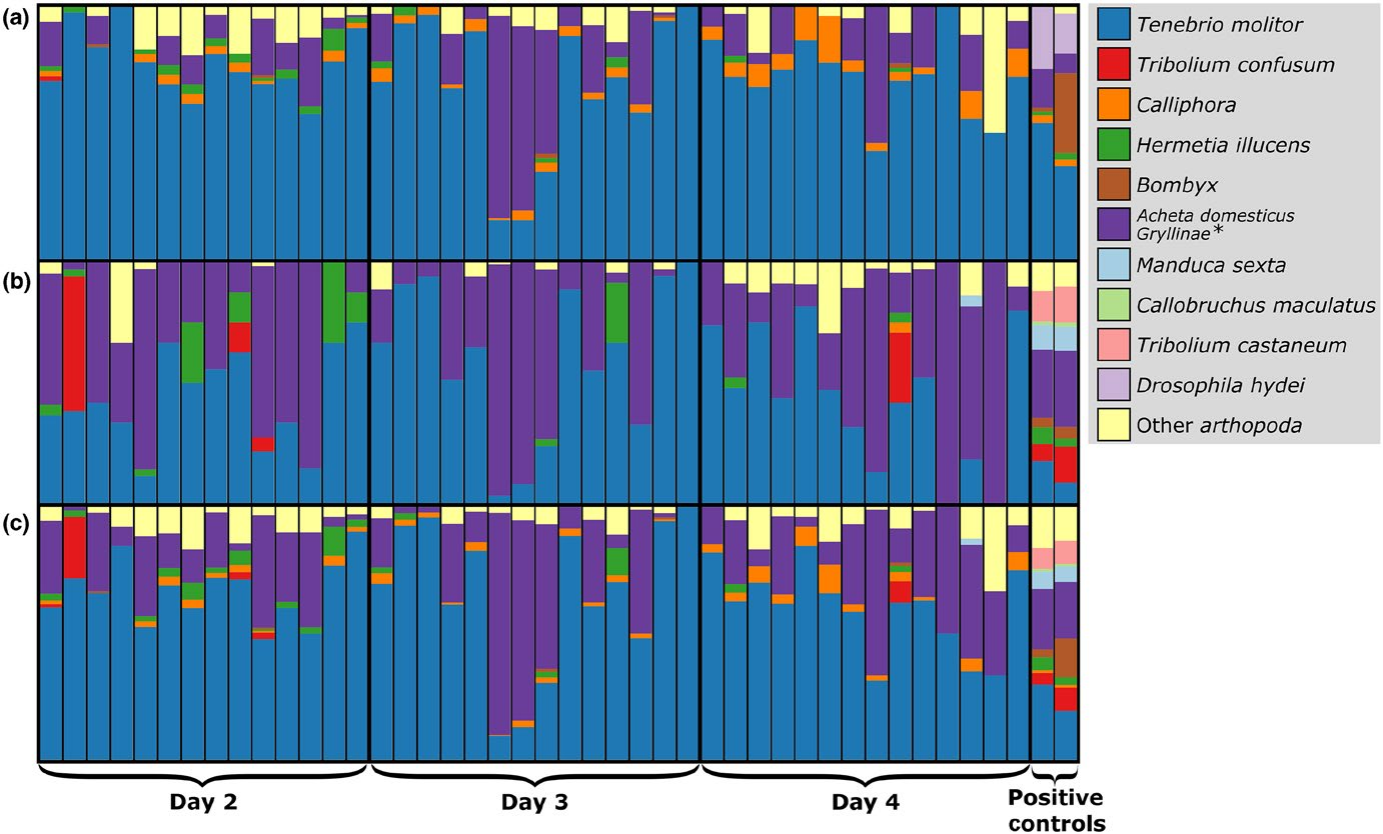
Proportional diet composition of the guano samples from the insectivorous *Antrozous pallidus* collected from the controlled feeding trial at the Fort Worth Zoo. Brackets and thick black lines separate collection days and positive controls, and thin lines separate individual samples. (a) *COI* genetic marker, (b) *16s* rRNA marker, and (c) combined results using both primers. *Gryllinae was the deepest taxonomic level assigned by the COI primer (a)

Using Sanger sequencing of positive control DNA for the different insect diet items, BLAST searches of the *16s* rRNA primer sequences detected 10 of 11 insect species, while BLAST searches of the *COI* primer sequences only detected eight. The lack of detection of some taxa is due either to lack of resolution of the marker or gaps in the NCBI database. For the NGS runs of these two primers, we analyzed fecal samples from both feeding trials and from mixed‐DNA positive controls (pooling equimolar concentrations of DNA extracts from all insect species). Across all samples, the number of reads produced by each primer per sample was similar; *16s* ranged from 3,415 to 134,515 reads per sample, whereas *COI* ranged from 1,669 to 117,889. Using NGS and BLAST searches of the positive control samples, the *16s* primer detected eight of the 11 food items, while *COI* only detected six (Figure 2a, b). When we combined the data from both primers, we were able to detect 10 of the 11 diet items, missing only *Chilecomadia* (Figure 2c). *16s* demonstrated greater taxonomic resolution than *COI* (e.g., *16s* recovered *Acheta domesticus*, whereas *COI* only identified the sample to the Gryllinae). With NGS of guano samples from the feeding trials, we consistently found *Tenebrio molitor*,* Acheta domesticus*,* Calliphora vomitoria, Hermetia illucens,* and *Tribolim confusum* in the *A. pallidus* diet (Figure 2c), which corresponded well with observations on *A. pallidus* feeding preferences (Fort Worth veterinary staff, pers. comm.). Negative controls at all stages had low numbers of unique sequences that matched to insects (*COI* mean = 6, *16s* mean = 5) relative to those found in positive controls (*COI* mean = 52, *16s* mean = 94) and guano samples (*COI* mean = 43, *16s* mean = 24). Replicate runs using MDM recovered similar read counts and results as with NGS (Supporting Information Table S4 and Figure 4).

**Figure 3:**
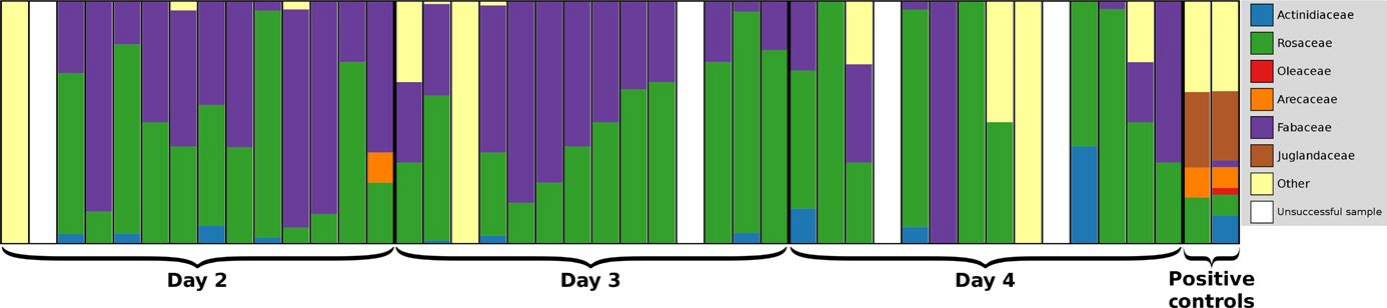
Proportional diet composition of the guano samples from the nectivorous *Leptonycteris yerbabuenae* collected from controlled feeding trial at the Fort Worth Zoo, using a primer targeting the plastid *trnH‐psbA* spacer region. Brackets and thick black lines separate the collection days and positive controls, and thin lines separate individual samples

**Figure 4:**
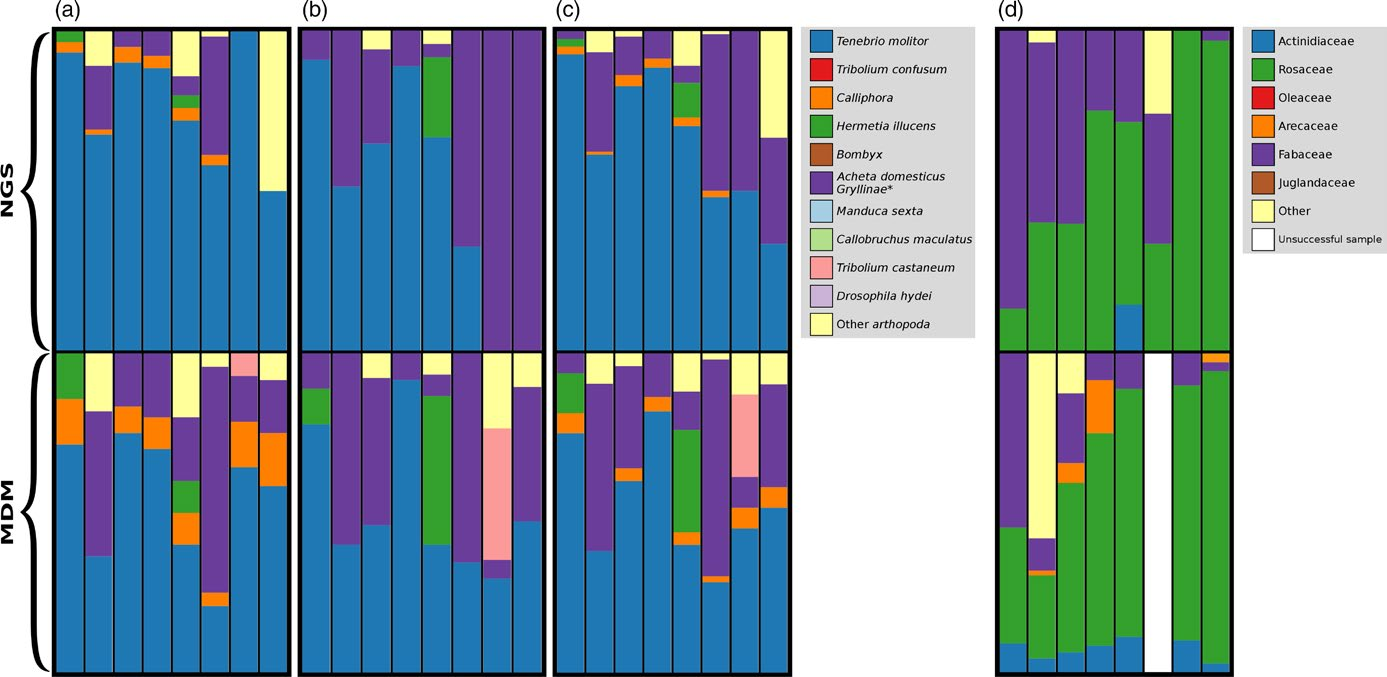
The repeatability of the identification of diet items between NGS and MDM. Results from (a) the *COI* marker for insectivorous diet, (b) the *16s* marker for insectivorous diet, (c) a combined analysis of the *COI* and *16s* markers for insectivorous diet, and (d) the *trnH‐psbA* marker for nectivorous diet

With direct Sanger sequencing of nectivorous bat diet items, BLAST searches of sequences resulting from the *ITS2, rbcL, trnL‐F,* and *trnH‐psbA* primers detected >80% of the items, with lack of detections reflecting poor sequence quality, poor resolution of the marker, or inadequacy of the NCBI database. With NGS testing, we found that most primers provided very few or poor‐quality sequences except for *trnH‐psbA*. Across all samples (both guano and positive control samples), this primer pair produced from 1,544 to 32,475 reads per sample. In the two positive control samples, which included the pollen from the plant families Rosaceae, Actinidiaceae, Arecaceae, and Oleaceae (but did not include the nectar mix with a soy base; Fabaceae), *trnH‐psbA* detected either two or four of the four plant families included in the pollen mix (Figure 3). With the 42 guano samples analyzed for the nectivorous diet feeding trials (that should have contained Fabaceae, Rosaceae, Actinidiaceae, Arecaceae, and Oleaceae), we detected Rosaceae (*n* = 34), Fabaceae (*n* = 31), Actinidiaceae (*n* = 10), and Arecaceae (*n* = 1), while Oleaceae was not detected. Unexpectedly, both positive controls showed multiple hits to Juglandaceae. Negative controls had a few unique sequences that matched to plants (mean = 2) relative to the positive controls (mean = 26) and guano samples (mean = 21). A replicate run of eight samples using MDM showed greater average read counts per sample and detected Fabaceae (*n* = 7), Rosaceae (*n* = 7), Arecaceae (*n* = 7), and Actinidiaceae (*n* = 5), but did not detect Oleaceae (Figure 4).

### 3.3 *Pd* detection

The samples of *M. lucifugus* that were identified as positive for WNS by USGS NWHC and verified to contain *Pd* using qPCR assays showed detections in all eight samples in the MDM run, with a read depth ranging from 9 to 17,324 (mean = 5,350). *Pd* was also detected in 13 negative samples, including one blank negative control sample, but with a much lower average read depth (range 6–20; mean = 12).

### 3.4 Parasite analysis

Endoparasites found in the necropsies of *Eptesicus fuscus* samples were categorized into three groups: Trematoda, Nematoda, and unidentified eggs (Table 4). Although we tested eight PCR primer pairs for nematode and trematode endoparasites targeting *COI* and *18s* rRNA, we found that one primer targeting *18s* provided the greatest accuracy (Table 2), and we therefore present only the results of this primer (Table 4). The NGS runs using the *18s* primer produced between 2,453 and 42,149 reads per sample. For the detection of trematodes, necropsies and NGS agreed in 12 of 16 samples (Table 4). For the detection of nematodes, NGS identified nematodes in nine samples, whereas necropsies found nematodes in only one sample (Table 4). NGS provided greater taxonomic resolution than the necropsies; while the parasites found in the necropsies could only be visually assigned to the phylum Nematoda or class Trematoda, trematodes detected with NGS/MDM were all assigned to the family Lecithodendriidae and nematodes were assigned across different taxonomic ranks, including the orders Tylenchida and Chromadorea, the family Capillariidae, and the genera *Fictor* and *Pristionchus*.

**Table 4:** Endoparasite detection results from necropsies and NGS analysis of 16 *Eptesicus fuscus* guano samples

Individual bat	Necropsy			NGS analysis	
	Trematoda	Nematoda	Eggs	Trematoda	Nematoda
1	+	+	+		1
2	+		+	1	1
3	+		+	4	3
4					
5	+		+	9	
6			+	1	
7	+		+	1	1
8	+		+		
9	+		+		1
10			+		
11			+		
12			+		2
13			+		2
14	+		+	2	1
15					1
16	+		+	8	

Necropsy results are separated into three classes (trematodes, nematodes, and eggs), with “+” representing presence. NGS results are indicated with the number of unique sequences annotated for nematodes and trematodes.

### 3.5 Species identification

Although we started with two primer cocktails to identify bat species (Zinck, Duffield, & Ormsbee, 2004; Lance et al., unpublished data; Supporting Information Table S2 #19–23), each consisting of two or three primer pairs targeting mitochondrial DNA regions, we found that, when used individually, none of these markers was able to consistently amplify in all species that we tested. However, NGS testing of the diet primers unexpectedly revealed that a single primer pair, the *16s* primer pair for the insectivorous diet, provided reliable amplification and was extremely accurate for determining the species of bat (Tables 2 and 5); we therefore present results using only the insect 16s diet primer for bat species ID (Tables 2 and 5). Of the 56 guano samples from known bat species, the *16s* primer pair accurately identified the species of 54 (Table 5). Read counts of accurately identified samples ranged from 504 to 35,667 (mean = 15,968) and were lower in the two inaccurately identified samples (mean = 495).

**Table 5:** Results of species identification tests using MDM and bat guano samples, including the number of accurate and inaccurate sample identifications, with mean read counts in parentheses

Species	*n*	Accurately identified	Inaccurately identified
*Corynorhinus rafinesquii*	24	24 (17,560)	0
*Myotis lucifugus*	8	7 (3,704)	1 (981)
*Antrozous pallidus*	8	8 (5,345)	0
*Leptonycteris yerbabuenae*	8	7 (25,648)	1 (8)
*Eptesicus fuscus*	8	8 (24,079)	0

### 3.6 Sex determination

We collected guano samples of individuals of known sex and then used them to determine the accuracy of several molecular approaches for sex identification (Table 6), including two sets of primer cocktails (either Korstian or XGXYC), both of which were scored using both NGS and by observing the presence of X/Y bands on agarose gels. Using NGS with the two‐marker systems resulted in sequencing depths of 3–13,155 reads per sample, and we attained a 72% scoring rate (27% “failed”). Of the samples scored using NGS, the sexes of 84% were accurately identified and 16% were misidentified (Table 6). Based on gel visualization, we attained a scoring rate of 90%, and of the scored samples, the sexes of 78% were accurately identified and 22% were misidentified (Table 6). Negative controls within the NGS analysis produced no annotated hits to the X or Y chromosome for either of the primer sets.

**Table 6:** Results of sex identification of 70 guano samples with gel visualization and MDM, along with primer set used in gel visualization

Species	NGS			Gel visualization			Primer^a^
	Correct	Incorrect	Failed	Correct	Incorrect	Failed	
*Eptesicus fuscus*	3	0	7	7	3	0	XGXYC
*Lasiurus borealis*	5	0	5	8	0	2	KZF
*Myotis austroriparius*	8	0	2	7	3	0	XGXYC
*Myotis grisescens*	6	3	1	5	5	0	XGXYC
*Myotis lucifugus*	6	3	1	6	0	4	XGXYC
*Myotis sodalis*	8	0	2	8	1	1	XGXYC
*Nycticeius humeralis*	7	2	1	8	2	0	KZF

a KZF, Korstian zinc finger; XGXYC, Xin Guan X and Y chromosome.

### 3.7 Genotype analysis

When using MicNeSs, two microsatellite loci, H09 and C04, showed inconsistencies in scoring because their repeat region is interrupted by a short span of nonrepeat sequence, the program can only score one portion of the microsatellite, and it may not consistently score the same portion across different samples or replicate NGS runs. Although we also used loci with compound repeats containing two unique repeat motifs (E07 and F02), these are amenable to scoring using MicNeSs because only one of the repeat motifs is scored.

NGS had lower percentages of missing data than conventional peak scoring on a genetic analyzer instrument (<1% vs. 15%, respectively), even though some samples were run up to three times on the genetic analyzer. Results from NGS exhibited lower allelic diversity than peak scoring, with a total of 32 and 80 alleles scored across all loci, respectively (mean = 5 vs. 13 alleles per marker, respectively). NGS and peak scoring resulted in similar values of observed heterozygosity (*H*_O_ = 0.669 and 0.655, respectively; Table 7), but differed for expected heterozygosity (*H*_E_ = 0.693 and 0.830, respectively; Table 7) and G_IS_ (inbreeding coefficient; 0.211 vs. 0.034, respectively; Table 7). A total of 77 unique genotypes were identified in the NGS data versus 88 in the peak scoring data (Table 7). The tests to determine repeatability using 23 individuals showed a repeatability rate of 90%. These errors were the result of MicNeSs identifying additional, erroneous alleles in low‐quality samples, which were generally outside of the size range observed for a microsatellite (e.g., six repeat units when the rest of the alleles are 20–30 repeat units).

**Table 7:** Results of analyses of genetic diversity and identity analysis for 94 samples of *Corynorhinus rafinesquii,* including mean number of alleles (*A)*, effective number of alleles (*A*_E_), observed heterozygosity (*H*_O_), expected heterozygosity (*H*_E_), inbreeding coefficient (*G*_IS_), number of unique genotypes (*n*_G_), and number of individual samples (*n*)

Dataset	*A*	*A*_E_	*H*_O_	*H*_E_	*G*_IS_	*n*_G_	*n*
Capillary	13	7	0.655	0.830	0.211	88	94
NGS	5	4	0.669	0.693	0.034	77	94

### 3.8 Overall success of the MDM approach

For the final MDM run, we obtained a total of 24,105,553 reads, and we were able to generate data from all six data classes simultaneously in a single NGS run. We generated data for the same samples and markers using both NGS (i.e., only a single data class) and MDM (Supporting Information Table S4) for the insectivorous and nectivorous diet and endoparasite assays. The average read count per sample differed between NGS and MDM replicates (Supporting Information Table S4), but was not consistently larger in either NGS or MDM. Despite the variation in the average read counts per sample, the repeated assays generally showed highly consistent results. For the eight samples of *E. fuscus* that were analyzed for endoparasites using both NGS and MDM, the samples identified as containing nematodes and trematodes were the same except for one sample that differed in whether nematodes were detected (Supporting Information Table S5). NGS and MDM also differed only slightly in the number of unique nematode or trematode sequences detected per sample (Supporting Information Table S5) despite the lower average read counts obtained for this assay in MDM (Supporting Information Table S4). For the two insectivorous diet markers, the average read counts per sample were comparable and the diet items identified were consistent across NGS and MDM (Supporting Information Table S4; Figure 4). For the nectivorous diet, the average read count per sample for MDM was more than double that found for NGS, and MDM consistently identified more of the items provided to the bats (Figure 4).

## 4 DISCUSSION

The goal of this study was to develop a rapid approach for simultaneously procuring information on bat populations while causing minimal stress to the bats. Although previous studies have used DNA metabarcoding to characterize a single type of information present in fecal samples, such as diet, few previous studies have used DNA metabarcoding to simultaneously assay multiple data classes present in fecal samples (but see De Barba et al., 2017). In this study, we demonstrated that NGS data could characterize bat species composition, individual genotype, sex ratios, prey, parasites, and pathogens. The accuracy of the PCR primers to quantify the data classes varied; below, we discuss each of the assays and their accuracy. We also showed that it was possible to obtain multiple classes of data from fecal samples using a combined NGS run (i.e., MDM), and preliminary data show that results are consistent whether we include assays for a single data class or multiple data classes.

### 4.1 The performance of each MDM assay

Knowledge of the diet of bats can provide insight into their ecology and species interactions and can help highlight the habitats or communities that should be protected to ensure that they have adequate food resources. For the insectivorous diet assay, we tested two primers (*COI* and *16s*), and we detected differences between them in the number of species that they could detect, caused by a lack of resolution of the marker or inadequacy of the NCBI database. Using MDM, we also found a lower number of detections for each primer relative to direct Sanger sequencing, likely caused by amplification biases in the mixed sample, which could possibly be remedied by optimization of PCR conditions. Nonetheless, we found that, using a combination of two primers (*COI* and *16s*), the MDM protocol was effective for detection of 10 of the 11 (91%) items in the positive control samples (Figure 2c). For the guano samples collected from *A. pallidus* in the controlled feeding trials (Figure 2), we consistently detected five diet items, which corresponded to dietary preferences observed by zoo staff. Although we also detected sequences from arthropod taxa other than those that were provided in the controlled feeding trials, it is possible that other species of arthropods were present in the zoo enclosures that were consumed by the bats; we are therefore unable to quantify fully the accuracy rate of this assay. That being said, the overall results of the study indicate that NGS and MDM approaches can quickly and efficiently characterize the diet of insectivorous bats with high taxonomic resolution and would likely represent considerable time savings relative to conventional microscopy‐based approaches (Burgar et al., 2014; Clare, Barber, Sweeney, Hebert, & Fenton, 2011; Vesterinen, Lilley, Laine, & Wahlberg, 2013). Unlike microscopy‐based counts, NGS/MDM approaches are currently unable to quantify the relative abundance of different prey items, which may eventually be mitigated by efforts to account for biases in PCR and differences in digestibility of prey (Thomas, Jarman, Haman, Trites, & Deagle, 2014).

The nectivorous diet assay showed lower sensitivity and resolution than the insectivorous diet assay. Although we tested several different commonly used plant DNA barcoding markers, the *trnH‐psbA* marker was the only one to consistently produce sequences across the samples. One explanation for the poor results from some markers is that we used a common set of PCR conditions to enable eventual multiplexing, but these conditions may not have been optimal for all markers. As markers are amplified individually using MDM, we therefore recommend using the optimal amplification conditions for each marker as reported in the literature, with additional optimization of PCR protocols as necessary, which would likely improve the detection rates using MDM.

For the nectivorous diet assay, although we were able to reliably recover sequences for nectivorous diet from the *trnH‐psbA* marker, it could only identify each pollen sample to the family level. Analyses of the positive controls detected all four of the plant families offered to the bats, and unexpectedly, also included hits to Juglandaceae. Because our pollen was sourced from a facility that specializes in supplying pollen of fruit and nut crops to commercial nurseries (The Pollen Bank; Bakersfield, CA), the pollen mixes may contain a small amount of airborne pollen from plants in the area; the pollen bank is located in an area where the English walnut, *Juglans regia*, is a major crop (Beede & Hasey, 1997). Unlike the positive controls, using the single‐assay NGS run, we consistently detected only two of the five plant food items from the controlled feeding in the guano samples of *L. yerbabuenae*. However, a replicate run of eight samples using MDM that had higher average read counts per sample consistently detected four of the five plant diet items in the guano samples. These results suggest that detection rates in the nectivorous diet assay may be affected by read depths. If so, it may be possible to increase detection rates in the nectivorous diet assay by adding proportionally more of this PCR product to the pool of amplicons to increase read depths. In both the NGS and the MDM runs, one plant family provided to the bats (Oleaceae) was not detected. One explanation for the lack of detection of this family is that the pollen may not have been equally distributed within the nectar mix and therefore may not have been consumed at high enough concentrations to be detected. Results indicate that not all plant species in a nectivorous bat’s diet may be detectable using MDM in its current form, but continued work to optimize protocols to enable the use of established plant DNA barcoding markers for MDM, as well as identifying additional DNA barcodes that vary in taxonomic coverage and resolution in plants would likely improve MDM for diet analysis in nectivores and herbivores.

Understanding whether *Pd*, the fungus causing white‐nose syndrome in bats, is present in a population is critical for ensuring its effective management. Using NGS and MDM, we detected *Pd* in each of the eight individuals of *M. lucifugus* confirmed to have *Pd*. We also found *Pd* sequences in 12 individuals that were asymptomatic for WNS and one blank negative control sample. Although it is possible that the *Pd* fungus could be present in low levels in individuals that were asymptomatic for WNS, because we detected a small number of sequences in the blank negative control sample, the most likely cause of these detections is cross‐contamination during PCR preparation, which can occur in low amounts when working with samples in a 96‐well plate format. Tag switching during DNA sequencing also cannot be ruled out (Esling, Lejzerowicz, & Pawlowski, 2015). As accurate detection of *Pd* is critical, we suggest the following steps to avoid cross‐contamination and ensure accurate results: (i) Each population should be processed (from DNA extraction to sequencing) independently to avoid cross‐contamination between populations differing in the presence of *Pd*, (ii) negative controls should be included at every step of sample processing to detect cross‐contamination from the laboratory, and (iii) samples with low read depths should be bioinformatically filtered out. Taking these precautions, MDM should provide accurate results on the presence of *Pd* in a population, with the advantage that it can be conducted while simultaneously collecting data about other attributes of the populations. If MDM unexpectedly detects *Pd* in a population that is thought to be negative or if important management decisions depend on the results, we recommend confirming these positive *Pd* detections with qPCR (following Muller et al., 2013). In addition, confirming a positive result in an independent laboratory to rule out laboratory contamination would provide unequivocal evidence of a positive detection. The drawback to double‐checking positive results is that it may be costly, but it may nonetheless be necessary to ensure that management activities are based on accurate information.

Understanding whether bat populations are affected by parasites is important because high parasite loads can affect the fitness of individuals, which can in turn affect overall population viability (Webber & Willis, 2016). MDM and necropsies largely found similar results for trematodes, whereas MDM identified nematodes in many more samples than necropsies. Increased detection using MDM may in part be due to its ability to resolve the identity of the eggs that were unidentified in the necropsies. MDM also showed increased taxonomic resolution of parasites, which can help clarify their origins; for example, all of the trematodes detected are in the family Lecithodendriidae, which are known bat parasites (Lotz & Font, 2008; McAllister, Bursey, & Robison, 2011), as is one of nematode families detected, Capillariidae (Santos & Gibson, 2015). Other nematodes detected using MDM are arthropod parasites (e.g., *Pristionchus* and Tylenchida), which may have been acquired through their prey (Herrmann, Mayer, & Sommer, 2006). Given that the MDM removes the need to sacrifice individual bats and provides greater resolution than necropsies, it is clearly an attractive option for surveys of endoparasite communities in bat populations.

Understanding the bat species present in a roost is one of the most basic, fundamental pieces of information necessary for managing the population. Bat species identification using guano and MDM also provided highly reliable results. Species identification using the *16s* rRNA marker in MDM agreed with visual identifications in 96% of the samples. The two samples that were misidentified showed far lower read counts than those that were accurately identified, suggesting that their sample quality was low and that identifications based on low read counts should be avoided. Results therefore show that MDM is a highly effective tool for the identification of bat species from guano samples.

Being able to identify the individual genotypes of the bats in a population using fecal samples and MDM has multiple applications, such as understanding population size, demographic parameters, as well as genetic diversity and structure. For the analysis of individual genotype, we observed some variation between the results obtained using MDM and capillary electrophoresis. While the observed heterozygosity was similar between the two approaches, the number of alleles, expected heterozygosity, and the number of individuals inferred from capillary electrophoresis were greater than those found using MDM. This is likely because scoring the fragment lengths of microsatellite loci includes variation in both repeat number and nonrepeat indels (Germain‐Aubrey, Nelson, Soltis, Soltis, & Gitzendanner, 2016), whereas MicNeSs (Suez et al., 2016) measures the number of repeats of a single motif in an individual. Thus, it is unsurprising that MDM shows lower variation because it is quantifying a single homologous source of variation rather than multiple possible sources of variation. To facilitate NGS genotyping of microsatellites, future studies should consider the following recommendations: (i) avoid using a locus with a single repeat motif that is interrupted (e.g., CA_6_…CA_10_), which currently cannot be scored reliably using current bioinformatic tools, (ii) select loci that can be sequenced entirely with a single‐direction read, and (iii) select microsatellites with tri‐ or tetra‐ nucleotide, noncompound repeat motifs to facilitate scoring. The present study demonstrates that it is possible to use MDM and fecal samples to conduct microsatellite genotyping, but a more comprehensive analysis using a greater number of fecal samples, very careful selection of microsatellite loci, and analysis of other genetic parameters, such as population size and genetic structure, would be useful to further validate this assay. Furthermore, given that software for calling microsatellites from NGS data has only recently been developed, and it is likely that allele calling could be improved by additional software development. A good example of a well‐validated study using NIS and NGS genotyping of microsatellites is De Barba et al. (2017), who used tissue and hair samples from brown bear (*Ursus arctos*) and showed the utility and reliability of using NGS for microsatellite genotype, albeit with a different approach to microsatellite scoring.

Robust and accurate sex identification of bats is vital for studying roost composition and function (e.g., maternity roosts), which can be important for management decisions. Shaw et al. (2003) developed a widely used sex identification marker system for mammals, but the amplicon is often >1 kb in length, which may not always be amplifiable in degraded samples (e.g., guano) or appropriate for the read lengths of most NGS platforms. We employed two sets of internal primers within this region in NGS (Korstian et al., 2013; Lance et al., unpublished data), each of which amplify in a different subset of bat species; thus, in situations where guano is sampled from a bat colony or population with multiple possible species present, we recommend using both primer cocktails in MDM to increase the probability of obtaining sex identification. Results of this study show 10%–27% failure rates for sex determination from guano samples using molecular approaches in bats; further optimization of PCR conditions could likely improve detection rates using both gel electrophoresis and NGS.

### 4.2 Overall strengths, weaknesses, and future directions of MDM

Metabarcoding may not uniformly detect all taxa within a group of organisms (Pompanon et al., 2012). This was particularly apparent for our characterization of nectivorous bat diet. Designing suitable metabarcoding primers is a careful balancing act; selecting regions that have stable primer‐binding sites often results in less variable sequences and lower taxonomic resolution, whereas primers targeting highly variable regions often have less stable primer‐binding sites and may not provide broad taxonomic coverage (Deagle, Jarman, Coissac, Pompanon, & Taberlet, 2014). In the MDM protocol, we selected as few markers as possible for each data class, which decreases the costs of molecular laboratory reagents, increases sequencing coverage, and streamlines bioinformatic analysis. In some cases, it may be advantageous to include multiple markers for a specific assay. For any metabarcoding study, we therefore recommend beginning with a broad range of primers with varying taxonomic resolution and optimizing PCR conditions to ensure high detection rates. Further, because the accuracy of the primers varied, we also recommend testing their accuracy before making real‐world management decisions based on the results.

Another step that may be customized to improve the accuracy of MDM assays for real‐world applications is filtering BLAST results. The samples in the present study were relatively low complexity and contained a limited number of known taxa; we therefore used the default settings in MEGAN6 to filter out low‐quality BLAST matches. The default settings in MEGAN6 are relatively liberal, with a maximum *E* value of 0.01 and no minimum percent identity. In real‐world situations where having a high confidence in BLAST annotations is important, customizing the *e*‐value threshold and minimum match percentage may be advantageous; settings would likely depending on the assay and how the data would be applied.

In all assays, as is typical of current NGS approaches, we detected a small amount of background contamination in negative controls. This may be particularly a problem in some assays, such as for *Pd*, where false positives may have important management consequences. Use of negative controls is therefore crucial, because no matter the level of laboratory sterilization and adherence to protocols, a low level of contamination is inevitable due to the acute sensitivity of PCR and NGS (King, Read, Traugott, & Symondson, 2008; Pompanon et al., 2012). Contaminant DNAs in negative controls for our laboratory processing steps (i.e., extraction and PCR blanks) typically exhibited very low read counts and can easily be filtered out in the bioinformatic pipeline. Contaminant DNAs found in negative controls from field collection surfaces (e.g., plastic sheeting under roosts) were present with higher read counts but were still low enough to effectively allow filtering. To handle contamination, we therefore recommend including negative controls throughout the process, strict processing conditions, data filtering based on minimum read counts, and, in situations where results have major management consequences, additional sample processing using independent assays.

Another factor not investigated here, but one that could potentially affect the results of metabarcoding analyses, is translocation of DNA segments, either from the mitochondria to the nuclear genomes within a species (i.e., nuclear mitochondrial DNA segment; or numts) or through horizontal gene transfer (HGT) between species, both of which are common in mammalian and insect genomes (Bertheau, Schuler, Krumböck, Arthofer, & Stauffer, 2011; Triant & DeWoody, 2007). Once they have translocated to the nuclear genome, numts experience different selection pressures and begin to accumulate differences relative to the functional mitochondrial copy; amplification of these fragments in metabarcoding may therefore lead to inaccurate species identifications or an overestimation of the number of taxa identified in a sample (Song, Buhay, Whiting, & Crandall, 2008). Likewise, horizontal gene transfer has been documented to occur from insect prey into the genomes of bats (Tang et al., 2015), which may cause inaccuracies in the estimation of diet composition using metabarcoding. Future work involving a detailed analysis of gene coding regions in metabarcoding data is necessary to investigate the extent to which numts and HGT may affect the identification of bat species and diet analysis using metabarcoding.

Some of the main advantages of MDM are its speed, ability to employ NIS, and, in most cases, high levels of accuracy. It also provides results that are very consistent with those obtained using single‐assay NGS. These attributes are likely to make it widely applicable and useful for understanding a diverse range of important attributes about bat populations. Also adding to its utility is the independence or modularity of the assays, such that new assays can be designed, validated, and incorporated into future MDM runs. Likewise, primers for data classes that are not needed or perform poorly can simply be excluded. Land managers can have the flexibility to choose the MDM assays to answer the most pertinent questions for a given population or species.

### 4.3 Future applications for MDM

This research demonstrates the potential utility of MDM to simultaneously investigate multiple aspects of the ecology of bats using a relatively noninvasive sampling technique. Because it is noninvasive, an ideal application of MDM would be to conduct repeated sampling over time to monitor changes in the status of populations. Genetic monitoring traditionally involves tracking changes in population genetic parameters such as heterozygosity or allelic richness, which can help detect whether species may be exhibiting negative trends such as losses in genetic diversity as the result of genetic bottlenecks, drift, or inbreeding. If genetic monitoring were implemented using fecal samples and MDM, it will be possible to use NIS to track not only genetic changes in populations, but also changes in many additional important parameters, such as diet or the presence of parasites and pathogens, which may be useful for interpreting changes in population sizes and for designing specific management strategies for populations.

Another positive attribute of MDM is its flexibility, as it is possible to add or delete specific assays depending on the needs of the user. Because of this flexibility, the MDM approach has broad applicability beyond the study system presented here. It could be directly applied to assess a wide range of data classes from fecal samples from any animal species and customized by substituting PCR primers that target specific attributes of the biology of each organism. The MDM protocol could also be applied to other types of organisms and samples; for example, it could be used to understand the diversity of bacteria, fungi, and plant species in soil samples, or of vertebrate, invertebrate, plant, and fungal taxa in water samples. Given the wide range in the accuracy rates of the primers used in the present study, we highly recommend that any new primers included in MDM are tested for accuracy using samples of known composition before the results are used to make management decisions.

## CONFLICT OF INTEREST

None declared.

## DATA ARCHIVING STATEMENT

All raw Illumina read data were deposited in the National Center for Biotechnology Information (NCBI) Sequence Read Archive (SRA) under BioProject accession PRJNA428365. Scripts for data processing are available at Github: https://github.com/Kenizzer/Bat_MDM.
