# Supplementary material for: Multifaceted DNA metabarcoding: Validation of a noninvasive, next‐generation approach to studying bat populations
Source: Evol Appl. 2018 May 31;11(7):1120–38. doi: 10.1111/eva.12644 (PMC6050187; doi:10.1111/eva.12644)
Supplement: Supplementary file 1 [file EVA-11-1120-s001.docx]

**Table S1.** Collection information and number (*n*) of bat fecal samples from each location used in this study.

| **Species** | **State** | **Location** | **Year** | ***n*** |
| --- | --- | --- | --- | --- |
| ***Eptesicus fuscus*** | IN | Crane Army Ammunition Activity | 2015 | 16 |
| ***E. fuscus*** | MS | Bridge #4, Rodney Rd. | 2015 | 5 |
| ***E. fuscus*** | NC | Fort Bragg | 2015 | 5 |
| ***Leptonycteris yerbabuenae*** | TX | Fort Worth Zoo | 2016 | 42 |
| ***Antrozous pallidus*** | TX | Fort Worth Zoo | 2016 | 42 |
| ***Lasiurus borealis*** | NC | Fort Bragg | 2015 | 10 |
| ***Myotis austroriparius*** | LA | Old Camp Claiborne | 2015 | 10 |
| ***M. grisescens*** | KY | Jesse James Cave | 2015 | 10 |
| ***M. lucifugus*** | N/A | USGS National Wildlife Health Center | 2009 | 10 |
| ***M. lucifugus*** | KY | Saltpeter Cave | 2015 | 5 |
| ***M. lucifugus*** | KY | B&O Cave | 2015 | 4 |
| ***M. lucifugus*** | KY | Mercers Cave | 2015 | 1 |
| ***M. sodalis*** | KY | Saltpeter Cave | 2015 | 10 |
| ***Nyctieius humeralis*** | MS | U.S. Army Engineer Research and Development Center | 2015 | 8 |
| ***N. humeralis*** | NC | Fort Bragg | 2015 | 2 |
| ***Corynorhinus rafinesquii*** | KY | Mammoth Cave National Park | 2015 | 94 |

**Table S2.** Primers tested within the study, with the most successful markers for each data class indicated with an asterisk in the first column.

| **#** | **Target group** | | **Forward Primer** | **Forward primer sequence** | **Reverse Primer** | **Reverse primer sequence** | **Region** | **Size**  **(bp)** | **Citation** |
| --- | --- | --- | --- | --- | --- | --- | --- | --- | --- |
| **1*** | Insects | ZBJ-ArtF1c | | AGATATTGGAACWTTATATTTTATTTTTGG | ZBJ-ArtR2 | WACTAATCAATTWCCAAATCCTCC | *COI* | 157 | (Zeale *et al.*, 2011) |
| **2*†** | Insects | Ins16S_1_F | | TRRGACGAGAAGACCCTATA | Ins16S_1_R | TCTTAATCCAACATCGAGGTC | *16s* | 216 | (Clarke *et al.*, 2014) |
| **3*** | Plants | psbAF | | GTTATGCATGAACGTAATGCTC | Trn-HR2 | CGCGCATGGTGGATTCACAAT | *TrnH-psbA* | 185-887 | (Sang, Crawford, & Stuessy, 1997) (Kress *et al.*, 2005) |
| **4** | Plants | ITS2-S2F | | ATGCGATACTTGGTGTGAAT | ITS2-S3R | GACGCTTCTCCAGACTACAAT | *ITS2* | 100-700 | (Yao *et al.*, 2010) |
| **5** | Plants | 390F | | CGATCTATTCATTCAATATTTC | 1326R | TCTAGCACACGAAAGTCGAAGT | *matK* | ~930 | (Cuénoud *et al.*, 2002) |
| **6** | Plants | 1F | | ATGTCACCACAAACAGAAAC | 724R | TCGCATGTACCTGCAGTAGC | *rbcL* | 724 | (Fay, Swensen, & Chase, 1997) |
| **7** | Plants | ITS1-F | | GATATCCGTTGCCGAGAGTC | ITS1-R | GGAAGTAAAAGTCGTAACAAGG | *ITS1* | >250 | (Baamrane *et al.*, 2012)  (White *et al.*, 1990) |
| **8** | Plants | g | | GGGCAATCCTGAGCCAA | h | CCATTGAGTCTCTGCACCTATC | *TrnL* | 12-134 | (Taberlet *et al.*, 2007) |
| **9** | Plants | Z1aF | | ATGTCACCACCAACAGACTAAAGC | 19bR | CTTCTTCAGGTGGAACTCCAG | *rbcL* | 110 | (Hofreiter *et al.*, 2000) |
| **10** | Plants | rbcL19a | | AGATTCCGCAGCCACTGCAGCCCCTGCTTC | rbcLZ1a | ATGTCACCACAAACAGAGACTAAAGCAAGT | *rbcl* | 153 | (Matheson *et al.*, 2008) |
| **11** | Plants | TA-s506 | | CGGGAGGTGGGGGAGAT | TA-a508 | TTTCCCTCTTCTTTGAGACMAG | *trnL* | 228 | (Staudacher *et al.*, 2011) |
| **12** | Plants | TrnL-e | | GGTTCAAGTCCCTCTATCCC | TrnF-f | ATTTGAACTGGTGACACGAG | *TrnL-TrnF* | 158-438 | (Taberlet *et al.*, 1991) |
| **13*** | Endoparasites | MN18F | | CGCGAATRGCTCATTACAACAGC | 22R reverse | GCCTGCTGCCTTCCTTGGA | *18s rDNA* | 345 | (Bhadury *et al.*, 2006) |
| **14** | Endoparasites | NemF3_t1 | | ARAGTTCTAATCATAARGATATTGG | NemR3_t1 | AAACCTCWGGATGACCAAAAAATCA | *COI* | 661 | (Prosser *et al.*, 2013) |
| **15** | Endoparasites | NemF2_t1 | | ARAGATCTAATCATAAAGATATYGG | NemR2_t1 | AWACYTCWGGRTGMCCAAAAAAYCA | *COI* | 661 | (Prosser *et al.*, 2013) |
| **16** | Endoparasites | 18S9F | | GATCCTGCCAGTAGTCATATGCTTG | 18S637R | TACGCTWYTGGAGCTGGAGTTACCG | *18s rDNA* | 600 | (Moszczynska *et al.*, 2009) |
| **17** | Endoparasites | LCO1490_t1 | | GGTCAACAAATCATAAAGATATTGG | HCO2198_t1 | TAAACTTCAGGGTGACCAAAAAATCA | *CO1* | 655-658 | (Folmer *et al.*, 1994) |
| **18** | Endoparasites | 18S9modF | | GATCCTGCCAGTAGTCATATGCTTG | 18S637modR | TACGCTWYTGGAGCTGGAGTTACCG | *18s* | 550-674 | (Moszczynska *et al.*, 2009; Modified by Van Steenkiste *et al.*, 2015) |
| **19** | Endoparasites | LCO1490 forward | | GGTCAACAAATCATCATAAAGATATTGG | chelicerate reverse1 | CCTCCTCCTGAAGGGTCAAAAAATGA | *CO1* | 660 | (Barrett & Hebert, 2005) |
| **20** | Endoparasites | chelicerate forward1 | | TACTCTACTAATCATAAAGACATTGG | chelicerate reverse2 | GGATGGCCAAAAAATCAAAATAAATG | *CO1* | 660 | (Barrett & Hebert, 2005) |
| **21*** | *P. destructans* | nu-IGS-0169-5’ | | TGCCTCTCCGCCATTAGTG | nu-IGS-0235-3’ | ACCACCGGCTCGCTAGGTA | Fungal *IGS* | 114-310 | (Muller *et al.*, 2013) |
| **22** | Species ID | Mysp1 | | GCACCTAGTTTACACCTAGAAGATT | Mtsp2 | TCTTTCATTTWTTCCCTTACGGTAC | *16s* | 190 | (Zinck, Duffield, & Ormsbee, 2004) |
| **23** | Species ID | Mysp3 | | AGAAGCAATAATGTTAATATGAG | Mysp4 | GCCGAGTTCCTTTTACTTCTTTT | *16s* | 190 | (Zinck *et al.*, 2004) |
| **24** | Species ID | BatCOI-P1-F | | AGGCACNGGTTGAACAGTAT | BatCOI-P1-R | HCCDGCAGCTAGGACTGGAA | *COI* | 20-206 | (Lance et al.,in prep.) |
| **25** | Species ID | BatCOI-P3-F | | TVATTGTCACTGCCCATGCT | BatCOI-P3-R | GCATGGGCDAGATTTCCTGC | *COI* | 59-247 | (Lance et al.,in prep.) |
| **26** | Species ID | BatCOI-P4-F | | GTNATCGTCACCGCCCATG | BatCOI-P4-R | GCATGGGCHAGATTTCCTGC | *COI* | 50-241 | (Lance et al.,in prep.) |
| **27*** | Sex primer | KXZF-F | | AGTCAAGGGRTGTCCATCR | KXZF-R | GTTTGYASACCAGGTTCCTC | Zinc finger X | 245 | (Korstian *et al.*, 2013) |
| **28*** | Sex primer | KYZF-F | | GGTRAGDGCACAYRAGTTCCACA | KYZF-R | TGCYATTACAAAACCTT | Zinc finger Y | 80 | (Korstian *et al.*, 2013) |
| **29*** | Sex primer | XGYC-F | | GCTGCTAAGCCACATATAGCT | XGYC-R | CCTGAATGTCTGTTCCAAAGACG | Zinc finger Y | 121 | (Lance et al.,in prep.) |
| **30*** | Sex primer | XGXC-F | | TGCGAGCTCTCAGATGAAACT | XGXC-R | TCCCTGTTCAATCCATTCCGT | Zinc finger X | 174 | (Lance et al.,in prep.) |
| **31** | Microsat | Cora_H09F | | ATTTATTCAGATGGAAATCAGCC | Cora_H09R | GAGTATCACCTACAGCCTCCTTT | Nuclear DNA | 110-132 | (Piaggio, Figueroa, & Perkins, 2009) |
| **32*** | Microsat | Cora_E07F | | TTACTAAAGGTTTGGGTAGGGAA | Cora_E07R | GTGAAGTAGCCTGGCCTAAGA | Nuclear DNA | 163-179 | (Piaggio *et al.*, 2009) |
| **33** | Microsat | Cora_F11 | | AAGCTCAGAGACTGCTCCTTC | Cora_C04 | ATCCATTATGTTTGCTGATGTTC | Nuclear DNA | 186-220 | (Piaggio *et al.*, 2009) |
| **34*** | Microsat | Cora_C07 | | CATTGGCTTTGTCTTAACAATTT | Cora_G01 | TTTGTTTCAGTTTCTCTCTCTCC | Nuclear DNA | 191-213 | (Piaggio *et al.*, 2009) |
| **35*** | Microsat | Cora_F02F | | GTCACTGGCTACAAAGAATGAAG | Cora_F02R | GAAACACAGCAGAATTGTCTCTC | Nuclear DNA | 201-263 | (Piaggio *et al.*, 2009) |
| **36*** | Microsat | Cora_G10 | | TTACAGTAGATACGGTTGTGCCT | Cora_A08 | TTTTAGGACTGGTTTTAGGGAAG | Nuclear DNA | 259-277 | (Piaggio *et al.*, 2009) |
| **37*** | Microsat | Cora_B07 | | TTAGACAAATGAGGGAGGATTG | Cora_H12 | CATCAAAGAATGCCAAACTAAAG | Nuclear DNA | 271-313 | (Piaggio *et al.*, 2009) |
| **38*** | Microsat | Cora_E10 | | ACTTTTCATTCTTTCCCATTCT | Cora G03 | AAACCAACGAGTGCTAAATCTAC | Nuclear DNA | 333-357 | (Piaggio *et al.*, 2009) |

† Indicates the insectivorous diet primer that can also be used for bat species identification.

**Table S3.** Itemized diet for *Leptonycteris yerbabuenae* at the Fort Worth Zoo used within the study.

| **Item** | **Unit** | **Fort Worth Diet** | **Transition Diet** | **Study Diet** |
| --- | --- | --- | --- | --- |
| Hot Water | G | 795 | 767 | 765 |
| Nekton Nectar Plus ( NEKTON-GmbH) | G | 164 | 112 |  |
| Fort Worth Bee Pollen Powder (In house mix) | G | 2 |  |  |
| Busy Bee Light Amber Honey (Barkman) | G | 56 | 26 |  |
| Calcium Carbonate | G | 2 |  |  |
| Controlled Pollen Mix (The Pollen Bank) | g |  | 2 | 2 |
| Nectar 3 (Roudybush) | g |  | 112 | 252 |
| Duration | days | na | 3 | 11 (7 :acclimatization & 4:collection) |

**Table S4.** The average read count for each type of markers across the samples repeated using NGS and MDM. The NGS run included only markers for a single data class whereas the MDM run contained multiple data classes. Numbers indicate the average number of reads per sample.

| **Assay** | **Species** | **Primer name** | **NGS read count** | **MDM read count** | ***n*** |  |
| --- | --- | --- | --- | --- | --- | --- |
| Nectivorous diet | *Leptonycteris yerbabuenae* | *trnH-psbA* | 10018 | 18284 | 8 |  |
| Insectivorous diet | *Antrozous pallidus* | *COI* | 30551 | 27242 | 8 |  |
| Insectivorous diet | *Antrozous pallidus* | *INS16s* | 37715 | 43252 | 8 |  |
| Endoparasites | *Eptesicus fuscus* | *MN18S* | 15661 | 1631 | 8 |  |

**Table S5.** Repeatability of endoparasite detection between NGS and MDM. The NGS run included only markers for endoparasites, whereas the MDM run contained multiple data classes. Results show the number of unique sequences annotated for nematodes and trematodes using the MN18F/22R primer pair in eight *Eptesicus fuscus* guano samples.

|  | **NGS** | | **MDM** | |
| --- | --- | --- | --- | --- |
| ***Eptesicus fuscus* individual** | **Trematoda** | **Nematoda** | **Trematoda** | **Nematoda** |
| 1 |  | 1 |  | 1 |
| 2 | 1 | 1 | 2 | 3 |
| 4 |  |  |  |  |
| 5 | 9 |  | 3 |  |
| 8 |  |  |  |  |
| 10 |  |  |  |  |
| 11 |  |  |  |  |

**Appendix S1: Supplementary Methods**

Sanger Sequencing of Diet Items

            All PCR reactions totaled 25uL in volume, consisting of 0.5 U of GoTaq Flexi DNA polymerase (Promega), 1× Promega Colorless GoTaq Flexi Buffer, 1.5 mM MgCl_2_, and 1 uM forward and reverse primers. PCR conditions were as followed: initial denaturation at 94 °C for 3 min, followed by 34 cycles of 94 °C for 30 sec, 52 °C for 30 sec, and 72 °C for 45 sec, and final extension at 72 °C for 20 min. Following PCR, samples were checked for amplification via gel electrophoresis and cleaned with EXOSAP (Exonuclease I and shrimp alkaline phosphatase; NEB). The mixture and samples were incubated at 72 °C for 45 min followed by 15 min at 80 °C.  Cleaned DNA was then quantified using Qubit (Life Technologies).  Forward and Reverse Sanger sequencing was conducted using BigDye chemistry on an ABI 3500 at the DNA analysis facility on Science Hill at Yale University. Returned chromatograms were visually inspected, edited, and assembled using Geneious R7 (Kearse et al. 2012). After which BLASTN searches were performed in Geneious against a local version of the NCBI Genbank nt database (downloaded 09/21/2016) using analogous settings to the command line BLASTN used within the other analyses.

Sex Identification Primer Development and Scoring

Using the LGL331 and LGL335 primers developed by Shaw et al. 2003 which flank the Zfx and Zfy introns located on the X and Y chromosomes. We verified that males produced two bands and females produced one band using gDNA extracted from wing pouch tissue and amplified according the conditions described in Shaw et al. 2003, for *Myotis grisescens* and *Eptesicus fusucs*. After which we cloned PCR products using TOPO TA pCR4.0 kit (Thermo Fisher Scientific). We successfully sequenced of the ZFx and ZFy introns from both species using M13 primers and BigDye Sequencing kit v3.1 (Invitrogen) and ABI 3500XL Genetic Analyzer (Applied Biosystems). Sequences were assembled and aligned using Geneious R8. Primers were designed in the Geneious R8 using Primer 3 (Untergasser *et al.*, 2012).

All PCRs were conducted in 20µl volumes containing; 1µl of template DNA (approximately 1-50ng/µl), 0.5 µM of each X-primer, 0.5µM of each Y-primer, 1X Qiagen Multiplex PCR Master Mix with HotStarTaq (Qiagen). The cycling parameters for PCRs were: one cycle at 95 °C for 15 min, followed by 30 cycles of 94 °C for 30 sec, 55 °C for 15 sec, 72 °C for 30 sec, and final extension at 72°C for 7min. PCR products were then run on 2% E-Gel® 48 Agarose Gels (Thermo Fisher).

qPCR detection of *Pseudogymnoascus destructans*

In order to verify that all *Myotis lucifugus* samples received from United States Geological Survey’s National Wildlife Health Center (Madison, WI, USA) were positive for White-Nose Syndrome (i.e. contained the fungus *Pseudogymnoascus destructans*) samples were subjected to qPCR using primers developed by Muller *et al.*, 2013, specifically nu-IGS-0169-5-Gd’ and nu-IGS-0235-3-Gd’. qPCR was run in 20 µL reaction volumes using 2X TaqMan^®^ Environmental Master Mix, 10 µM of each primer, 0.25 µM of the probe, and 1µL of DNA template. Reactions were run on a ViiA™ 7 Real-Time PCR System (Applied Biosystems) using the Standard protocol for Presence/Absence. The cycling program began with initial denaturation at 95°C for 10 min, followed by 40 cycles of 95°C for 15 sec and 60°C for 1 min. Each of the WNS-positive samples was run six times to confirm the presence of *P. destructans* in the guano sample, with six water blanks included as negative controls.

Conventional Microsatellite Genotyping (Capillary Electrophoresis)

We evaluated 15 microsatellite loci previously developed for *Corynorhinus rafinesquii* (Piaggio et al. 2009) using DNA extracted from guano samples collected from beneath the manmade *C. rafinesquii* roost at Mammoth Cave National Park (KY, USA). Of these loci, 8 successfully and consistently amplified *C. rafinesquii* DNA from guano and were found to be polymorphic, including A08, C04, E07, F02, G01, G03, H09, and H12. All loci contained dinucleotide repeat motifs. M13 primer sequences were added to the 5’ end of each *C. rafinesquii* primer for use with M13 primers labelled with FAM or HEX to multiplex during fragment analysis. We conducted PCR in 10 µL reactions with 0.15 µm forward primer, 0.3 µm reverse primer, 0.45 µm labeled M13 primer, 0.2 mM each dNTP, 2 mM MgCl_2_, 0.2 µm BSA, 1 U Platinum Taq polymerase, 1X buffer, and 10-20 ng gDNA. Negative controls were included in all amplifications and reactions were prepared in a laminar flow PCR hood in a separate room from the extractions. Annealing temperature was 52°C for all primers except E04, which was 54°C. Amplification with msDNA primers was conducted with initial denaturation at 94°C for 10 min, followed by 40 cycles of 94°C for 30 sec, and 72°C for 45 sec, followed by a 30 min extension at 60°C. Fragment analysis was carried out on a 3500 XL Genetic Analyzer with GENEMAPPER software (Applied Biosystems, Grand Island, NY) and LIZ 500 size standard. Fragment analysis reactions consisted of 1 μL each PCR product, 0.5 μL size standard, and HiDi Formamide (Applied Biosystems) to 12.5 μL total volume. All putative homozygotes and failed samples were amplified and genotyped at each locus a minimum of three times to confirm their status.
